# Anti-Proliferative Effects of HBX-5 on Progression of Benign Prostatic Hyperplasia

**DOI:** 10.3390/molecules23102638

**Published:** 2018-10-14

**Authors:** Bo-Ram Jin, Hyo-Jung Kim, Sang-Kyun Park, Myoung-Seok Kim, Kwang-Ho Lee, Il-Joo Yoon, Hyo-Jin An

**Affiliations:** 1 Department of Pharmacology, College of Korean Medicine, Sangji University, 83 Sangjidae-gil, Wonju-si, Gangwon-do 26339, Korea; wlsqh92@gmail.com (B.-R.J.); hyojung_95@naver.com (H.-J.K.); 2 Department of Meridian & Acupoint, College of Korean Medicine, Sangji University, 83 Sangjidae-gil, Wonju-si, Gangwon-do 26339, Korea; psk7509@sangji.ac.kr; 3 Central Research Institue of Hawon Pharmaceutical, Jangheung 59338, Korea; mskim1210@naver.com (M.-S.K.); gusins@hanmail.net (K.-H.L.); polorman@naver.com (I.-J.Y.)

**Keywords:** BPH, HBX-5, proliferation, inflammation, RWPE-1, WPMY-1, testosterone-induced BPH rat model

## Abstract

Benign prostatic hyperplasia (BPH), an age-dependent disorder with a prevalence percentage of 60% in the 60s, has been found to involve an androgenic hormone imbalance that causes confusion between cell apoptosis and proliferation. Because general medications for BPH treatment have undesirable side effects, the development of effective alternative medicines has been considered. HBX-5 is a newly developed formula with the aim of improving BPH, and is composed of nine medicinal herbs. BPH was induced in the rats by intramuscular injection of testosterone propionate after castration. Rats were divided into six groups, and the efficacy of HBX-5 on testosterone-induced BPH in rats was estimated. In addition, RWPE-1 and WPMY-1 cells were used to demonstrate the effect of HBX-5 on BPH in vitro model. Compared with the control group, HBX-5 administration group suppressed BPH manifestations, such as excessive development of prostate, and increase of serum dihydrotestosterone and 5α-reductase concentrations. Furthermore, immunohistochemistry analysis revealed that HBX-5 significantly decreased the expression of androgen receptor (AR) and proliferating cell nuclear antigen (PCNA). In addition, results of RWPE-1 and WPMY-1 cells showed that HBX-5 inhibited the over-expression of AR and PSA in DHT-induced prostate hyperplastic microenvironments.

## 1. Introduction

Benign prostatic hyperplasia (BPH) is a nonmalignant growth condition, characterized by prostate enlargement, and accompanied by lower urinary tract symptoms (LUTS) and bladder outlet obstruction (BOO). Although BPH is uncommon before age 40, roughly, 50% of men develop BPH-related symptoms at 50 years of age. The incidence of BPH increases by 10% per decade and reaches to 80% at approximately 80 years of age [1,2]. Despite many decades of intensive research, the etiology of BPH still remains to be unraveled in some aspects. Of the dominant hypotheses, age-dependent changes and hormonal disturbances are mostly involved in the pathogenesis and progression of BPH [3]. Recently, prostatic inflammation has been focused to be associated with prostatic enlargement and symptomatic progression. There is notable evidence that chronic inflammation was found in 75% of prostates examined in comparison to 55% of normal group in the autopsy specimens obtained from 93 men with histological BPH [4]. The chronic inflammatory condition may contribute to tissue injury, which can induce compensatory cellular proliferation. These events represent disruption in the balance between cell death and cell proliferation, and then increase BPH [5].

Current medical modalities aimed at treating BPH include pharmacotherapy targeting 5α-reductase and α1-adrenergic receptors. The most frequently used 5α-reductase inhibitors are finasteride and dutasteride that inhibit the production of dihydrotestosterone (DHT). α1-adrenergic blockers reduce the binding of adrenergic hormone on cognate receptors and decrease smooth muscle tone of bladder neck [6,7]. Unfortunately, these therapies are not completely effective because side effects of this therapy include dizziness, weakness, insomnia, and high risk of sexual dysfunction, including erectile dysfunction, and ejaculatory disorders. As part of the efforts to reduce side effect and develop a nontoxic medicine, there has been an increasing interest in the development of herbal medicines. Herbal medicines have been the subject of many classic studies, because of appreciable treatment effects for various diseases and great accumulation of empirical experiences.

HBX-5 is a newly combined formula: 30% ethanol extract for treating BPH, composed of nine medical herbs (*Aconiti Lateralis* Radix Preparata, *Cornus officinalis*, *Cistanche salsa*, *Psoralea corylifolia* L., *Dendrobium nobile* Lindley, *Morinda officinalis* Haw, *Cuscuta japonica*, *Trigonella foenumgraecum* L., and *Foeniculum vulgare* Mill). Previous studies have reported *Aconiti Lateralis* Radix Preparata has noticeable anti-inflammatory effects [8]. *Cornus officinalis* has been used in the treatment of sexual dysfunction based on the traditional effects and cornuside, which is isolated from the fruits of *Cornus officinalis*, has vascular relaxation effects, indicating an improvement of sexual function [9]. In addition, our previous study demonstrated that the *Cistanche salsa* extract ameliorates the development and progression of BPH [10]. *Psoralea corylifolia* Linne has been investigated to induce spermatogenesis in rat testes and rehabilitate male reproductive functions [11]. The previous finding revealed that glycosides from Dendrobium nobile have an immunomodulatory activity [12]. *Morinda officinalis* has also been used to improve the sperm motility [13]. *Cuscuta japonica* is well-known to strengthen the generative function in east Asia and has anti-oxidant effects [14]. In addition, *Trigonella foenumgraecum* Linn. has traditionally been used as an important medicine in India and Africa, and has been investigated for its pharmacological properties, including antidiabetic, antioxidative, and anti-inflammatory effects [15]. *Foeniculum vulgare* Mill, commonly called fennel, has been used in traditional medicine and is reported to treat diuresis [15]. Furthermore, our group has also assessed the individual constituents of HBX-5 on animal with BPH model and prostate cell line. Based on their traditional uses and scientific evaluation, we explore the activities of HBX-5 under BPH condition, with the aim of identifying the molecular mechanisms that are associated with inflammation, apoptosis, and hyperplasia.

## 2. Results

### 2.1. Optimization of Chromatographic Conditions

**Figure 1:**
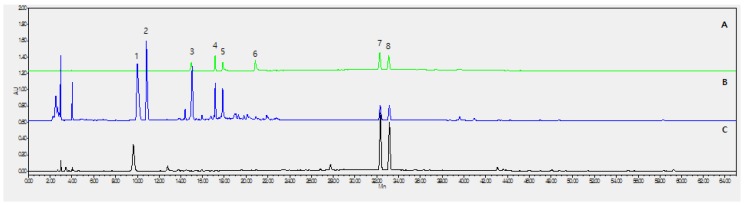
HPLC profile of HBX-5. (**A**) Mixed standards; (**B**) before acid hydrolysis; (**C**) after acid hydrolysis. (**1**) psoralenoside; (**2**) isopsoralenoside; (**3**) morroniside (**4**) loganin (**5**) echinacoside (**6**) verbascoside (**7**) psoralen; (**8**) isopsoralen.

**Figure 2:**
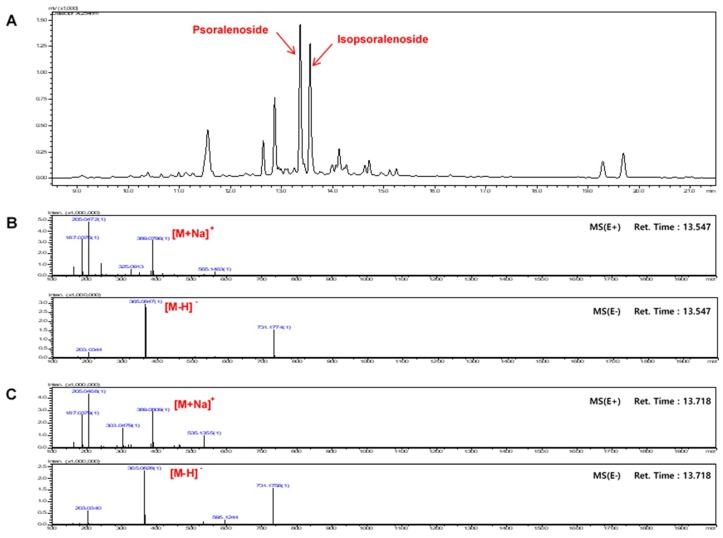
Ion chromatogram of HBX-5. (**A**) Total ion chromatogram and (**B**) MS spectra of psoralenoside (**C**) isopsoralenoside in HBX-5.

As shown in Figure 1, the 8 analytes had very broad range of polarity; therefore, gradient elution was utilized, and various mobile phase compositions were tested. The mixture of 10 mM ammonium acetate (*w*/*v*) aqueous acetic acid and acetonitrile was finally chosen as the preferred mobile phase, because it yielded the desired separation and acceptable tailing factors within the run time (70 min). Based on the ultraviolet absorption characteristics of the major compounds, the chromatograms were recorded at a wavelength of 290 nm. It was found that the separation was optimized when the column temperature was kept at 35 °C. Representative chromatograms of the mixed standards and the extract are included in Figure 1. The persistent high content of psoralen and isopsoralen could be explained by the acid hydrolysis of psoralenoside and isopsoralenoside, respectively. Figure 2 Shows total ion chromatograms (TIC) and mass spectrums (MS) of psoralenoside and isopsoralenoside of HBX-5.

### 2.2. Calibration Curves, Limits of Detection, and Quantification

The stock solution of the six reference compounds was diluted to a range of appropriate concentrations for the construction of calibration curves. Calibration curves were constructed by plotting the mean peak areas versus the standard concentrations. All calibration curves showed good linear regression (*r* ≥ 0.9950) over the tested ranges (Table 1). LOD and LOQ were separately determined at a S/N of 3 and 10, respectively.

### 2.3. Effect of HBX-5 Administration on Prostate Weight in Rat with BPH Models

The testosterone-induced rat with BPH was used to explore the study about the development of obstruction in micturition and evaluation of drugs targeting the BPH problems [16,17].

**Figure 3:**
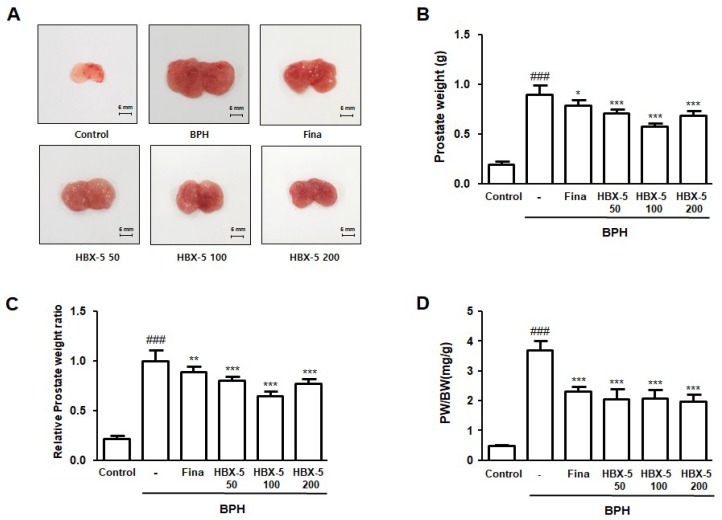
Effect of HBX-5 on prostate weight and prostate index in rat with BPH models. (**A**) A representative photograph of prostate in each group is provided. Change in (**B**) total prostate weight of the rat, (**C**) relative prostate weight ratio and (**D**) PW/BW ratio was assessed for the Control, rat with BPH, Fina and HBX-5 groups. Values are the mean ± SD (*n* = 10); ^###^ *p* < 0.001 vs. control group; * *p* < 0.05, ** *p* <0.01, *** *p* < 0.001 vs. the rat with BPH group; significances between treated groups were determined using ANOVA and Dunnett’s post hoc test.Control, Control group; BPH, rats with BPH group; Fina, rats with BPH group treated with 5 mg/kg/day of finasteride; HBX-5 50, rats with BPH group treated with 50 mg/kg/day of HBX-5; HBX-5 100, rats with BPH group treated with 100 mg/kg/day of HBX-5; HBX-5 200, rats with BPH group treated with 200 mg/kg/day of HBX-5.

Initially, we documented the typical macroscopic parameter of BPH, such as the swollen prostatic tissue and increased blood vessels. Figure 3A shows the representative prostate tissues of each group for visual comparison. The appearance of prostate in the BPH group showed significant prostatic congestion and enlargement of prostate tissue in comparison to the rats of all other groups. The mean prostate weight of the rats of the BPH group also increased significantly compared to the Control group (Figure 3B). In addition, the relative prostate weight ratio in the BPH group was 4.65 times higher than that in the Control group. In the finasteride-, HBX-5 50-, HBX-5 100-, and HBX-5 200-treated groups, the relative prostate weight ratio were 4.11, 3.70, 2.98, and 3.56 times higher than that of the control, respectively (Figure 3C). As shown in Figure 3D, PW/BW ratio was also significantly higher in the BPH group than that in the other groups.

### 2.4. Effect of HBX-5 Administration on the Histological Alteration in Rats with BPH

**Figure 4:**
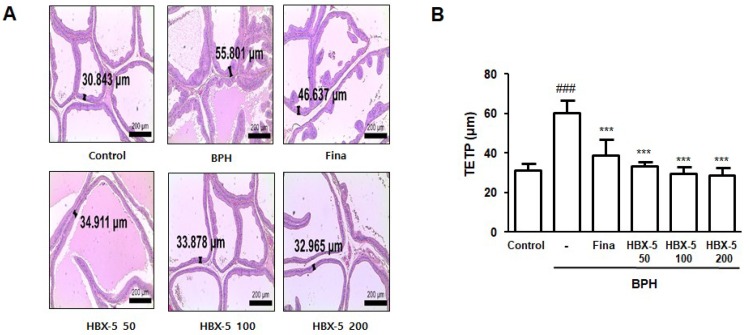
Effect of HBX-5 on the prostatic cell proliferation. (**A**) Hematoxylin and eosin staining of prostate tissue from rats with BPH models; original magnification 40×. (**B**) Thickness of prostatic epithelial tissue. TETP was measured and expressed as the mean ± standard error of 5 rats per group. TETP was quantified at three section per sample. Values are the mean ± SD (*n* = 10); ^###^ *p* < 0.001 vs. Control group; *** *p* < 0.001 vs. BPH group. significances between treated groups were determined using ANOVA and Dunnett’s post hoc test. Control, Control group; BPH, rats with BPH group; Fina, rats with BPH group treated with 5 mg/kg/day of finasteride; HBX-5 50, rats with BPH group treated with 50 mg/kg/day of HBX-5; HBX-5 100, rats with BPH group treated with 100 mg/kg/day of HBX-5; HBX-5 200, rats with BPH group treated with 200 mg/kg/day of HBX-5.

To investigate the effect of HBX-5 on testosterone-induced morphological changes of the prostate gland, we conducted an H & E staining analysis. As shown in Figure 4, each group showed a considerable gap in the hyperplastic patterns, such as an epithelium thickness and contraction of glandular luminal area. In the BPH group, a significantly thickened epithelium and reduced glandular luminal area was observed in comparison to the Control group. However, finasteride-, HBX-5 50-, HBX-5 100-, and HBX-5 200-treated groups significantly ameliorated the extent of testosterone-induced prostate hyperplasia morphology.

### 2.5. Effect of HBX-5 Administration on the Expression of AR, Type-2 5α-Reductase mRNA Expression, and Serum DHT Levels

**Figure 5:**
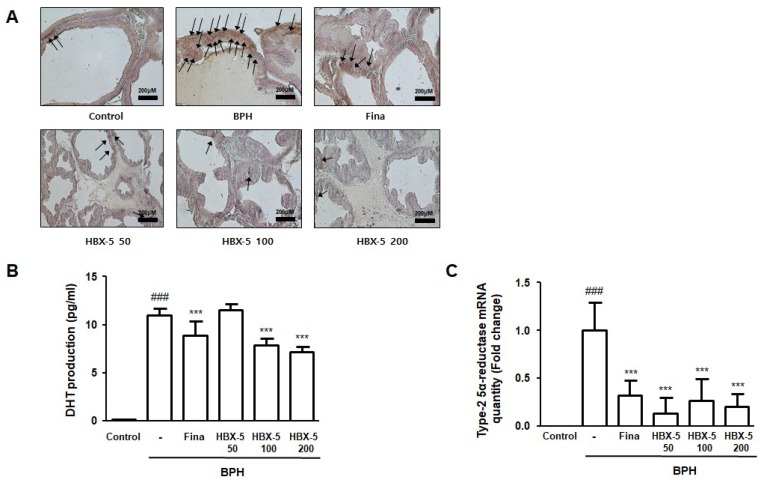
Effect of HBX-5 administration on the expression of AR and level of 5α-reductase 2 mRNA and production of DHT in rats with BPH. (**A**) The manifestation of AR in prostate tissues was performed by IHC. The arrows indicate immunoreactivity of AR in the representative pictures of each group. (**B**) Serum concentration of DHT was determined by ELISA. (**C**) Expression of 5α-reductase 2 mRNA in prostate tissues was analyzed by quantitative real-time PCR. Values are the mean ± SD (*n* = 10); ^###^ *p* < 0.001 vs. Control group; *** *p* < 0.001 vs. BPH group. significances between treated groups were determined using ANOVA and Dunnett’s post hoc test. Control, Control group; BPH, rats with BPH group; Fina, rats with BPH group treated with 5 mg/kg/day of finasteride; HBX-5 50, rats with BPH group treated with 50 mg/kg/day of HBX-5; HBX-5 100, rats with BPH group treated with 100 mg/kg/day of HBX-5; HBX-5 200, rats with BPH group treated with 200 mg/kg/day of HBX-5.

It is well known that androgen and AR signaling has a major influence in enhancing cell growth in both stromal and epithelial cells, which promotes the development of BPH [18]. The mechanism of androgen/AR signaling could be attributed to the involvement of 5α-reductase 2 and DHT production. Several studies have been addressed that testosterone is converted to DHT by 5α-reductase. DHT in serum reflects the activity of type-2 5α-reductase in the prostate [19]. In this study, we investigated whether HBX-5 inhibits the expression of AR, type-2 5α-reductase, and DHT production, thereby preventing the progression of BPH. It was observed that treatment with HBX-5 suppressed the abnormal expression of AR in rats with BPH, and finasteride treatment had similar effects (Figure 5A). In addition, HBX-5 100-, 200- and finasteride-treated groups were found to significantly decrease DHT production compared to BPH group. (Figure 5B). To find out whether this effect of HBX-5 was associated with the activity of type-2 5α-reductase, we examined the level of type-2 5α-reductase mRNA in prostatic tissues. The type-2 5α-reductase mRNA level in the BPH group was increased in comparison to the Control group. However, HBX-5 and finasteride significantly inhibited the activity of the enzyme (Figure 5C).

### 2.6. Effect of HBX-5 Administration on Prostatic Cell Proliferation

Disruption of balance between cell death and cell proliferation underline the abnormal growth of prostate gland, leading to BPH [20]. 

**Figure 6:**
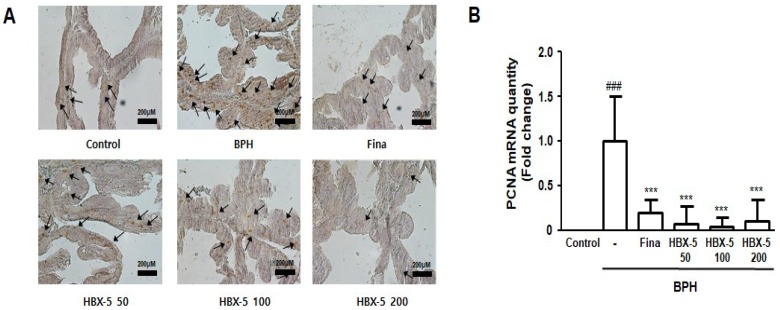
Effect of HBX-5 administration on the expression of PCNA. (**A**) The manifestation of PCNA in prostate tissues was performed by IHC staining. The arrows indicate immunoreactivity of PCNA in the representative pictures of each group. (**B**) Expression of mRNA for PCNA in prostate tissues was analyzed by quantitative real-time PCR. The data presented as the mean ± standard error of the mean of 10 rats per group (^###^ *p* < 0.001 vs. Con group; *** *p* < 0.001 vs. BPH group). Control, Control group; BPH, rats with BPH group; Fina, rats with BPH group treated with 5 mg/kg/day of finasteride; HBX-5 50, rats with BPH group treated with 50 mg/kg/day of HBX-5; HBX-5 100, rats with BPH group treated with 100 mg/kg/day of HBX-5; HBX-5 200, rats with BPH group treated with 200 mg/kg/day of HBX-5.

Proliferating cell nuclear antigen (PCNA) is considered to reflect cell proliferation, particularly, growth fraction; and previous studies reported that tissues from the patients with BPH and BPH animal models showed increased expression of PCNA. In this study, we conducted IHC staining and evaluated the extent of labeling to investigate the effect of HBX-5 on the expression of PCNA in rats with BPH. The expression of PCNA was higher in the BPH group than that in the Control group, and finasteride- and HBX-5-treatment groups showed moderate expression (Figure 6A). Furthermore, we also confirmed the mRNA level of PCNA, as detected by qRT-PCR. As shown in Figure 6B, PCNA mRNA level in the BPH group was increased in comparison to the Control group, and finasteride- and HBX-5-treatment groups exhibited a significant decrease in the PCNA mRNA level.

### 2.7. Effect of HBX-5 on the Expression of COX-2 and iNOS in the Prostatic Tissues of Rats with BPH Models

**Figure 7:**
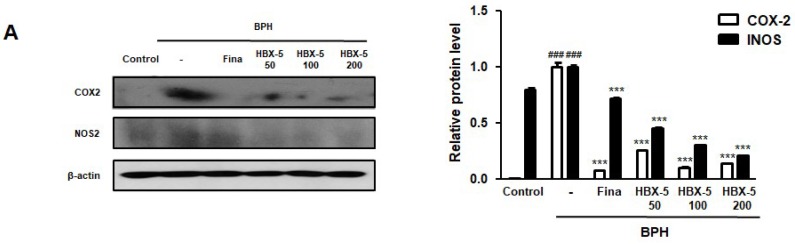
Effect of HBX-5 on the expression of COX-2 and iNOS in prostate tissues of rats with BPH models. (**A**) The protein levels of COX-2 and iNOS were determined by western blotting using specific antibodies. β-actin was used as an internal control. **(B)** Densitometric analysis was performed using ImageJ Software. The data presented as the mean ± standard error of the mean of 10 rats per group (^###^ *p* < 0.001 vs. Control group; *** *p* < 0.001 vs. BPH group). Control, Control group; BPH, rats with BPH group; Fina, rats with BPH group treated with 5 mg/kg/day of finasteride; HBX-5 50, rats with BPH group treated with 50 mg/kg/day of HBX-5; HBX-5 100, rats with BPH group treated with 100 mg/kg/day of HBX-5; HBX-5 200, rats with BPH group treated with 200 mg/kg/day of HBX-5.

The etiology of BPH is multifactorial, and chronic inflammation has been focused on more during its progression. Several studies have reported that tissues from BPH patients and animal models contained certain amount of lymphocyte-derived cytokines, which stimulated cell proliferation [21]. In addition, the overexpression of NF-κB/p65, COX-2, and iNOS has been confirmed in the testosterone-induced BPH model [22]. As shown in Figure 7, the BPH group showed higher expression of COX-2 and iNOS in comparison to the Control group, and the elevated expressions of these inflammatory proteins was down-regulated by HBX-5 and finasteride treatment.

### 2.8. Effect of HBX-5 Administration on the Expression of Apoptosis-Related Proteins in the Prostatic Tissues of Rats with BPH Models

**Figure 8:**
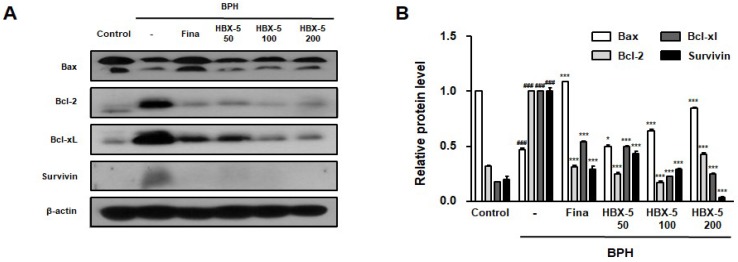
Effect of HBX-5 administration on the expression of apoptosis-related proteins in prostate tissues of rats with BPH models. (**A**) The expression levels of Bax, Bcl-2, Bcl-xL and Survivin were determined by western blotting using specific antibodies. β-actin was used as internal control. (**B**) The protein band densities were determined by densitometric analysis. The data showed representative mean ± standard error of the mean of 10 rats per group (^###^ *p* < 0.001 vs. Control group; *** *p* < 0.001 vs. BPH group). Control, Control group; BPH, rats with BPH group; Fina, rats with BPH group treated with 5 mg/kg/day of finasteride; HBX-5 50, rats with BPH group treated with 50 mg/kg/day of HBX-5; HBX-5 100, rats with BPH group treated with 100 mg/kg/day of HBX-5; HBX-5 200, rats with BPH group treated with 200 mg/kg/day of HBX-5.

It has been argued that the potential involvement of anti-apoptosis proteins in growth imbalance ultimately promotes prostate hypertrophy. Previous studies have reported that increased expression of Bcl-2, a potent apoptosis suppressor, was detected in BPH specimens in comparison to normal prostate specimens [20]. The results of this study corroborated with the findings of previous studies, which depicted that testosterone-induced BPH might be associated with the alterations in the Bax-to-Bcl-2 balance [23]. In Figure 8, HBX-5- and finasteride-treated groups showed decreased expression of Bcl-2, Bcl-xL, and Survivin; and increased expression of the pro-apoptotic protein, Bax. These findings suggest that HBX-5-induced apoptosis plays a critical role in the alleviation of BPH.

### 2.9. Effect of HBX-5 Administration on the BPH-Related Protein Expression in RWPE-1 and WPMY-1 Cells

**Figure 9:**
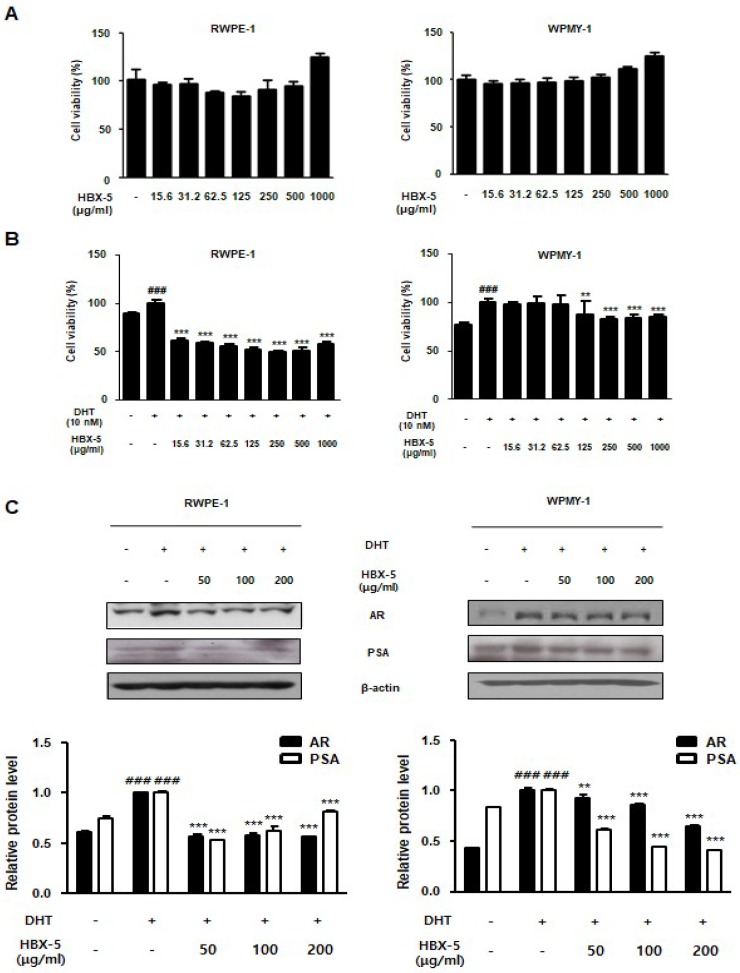
Effect of HBX-5 on the expression of BPH-related protein in RWPE-1 cells and WPMY-1 cells. (**A**) Effect of HBX-5 on the cell viability in RWPE-1 cells and WPMY-1 cells. RWPE-1 cells and WPMY-1 cells were treated with 15.6–1000 μg/mL of HBX-5 for 24 h. (**B**,**C**) The normal human prostatic epithelial cell line, RWPE-1, and normal human prostatic stromal cell line, WPMY-1, were treated with 10 nM DHT for 72 h and 24 h respectively, with or without HBX-5 (50–1000 μg/mL). (**B**) Inhibitory effect of HBX-5 on cell proliferation in DHT-stimulated prostate cells. (**C**) Anti-BPH effect of HBX-5 in DHT-stimulated prostate cells. The expression levels of AR and PSA were determined by western blotting using specific antibodies. β-actin was used as an internal control. The protein band densities were determined by densitometric analysis. Values represent mean ± S.D. of three independent experiments. (^###^ *p* < 0.001 vs. Control group; *** *p* < 0.001 vs. DHT-stimulated group).

The normal prostate epithelial cell RWPE-1 and stromal cell-line WPMY-1 were used for generating an in vitro BPH model. To confirm the effect of HBX-5 on the cell viability, we performed MTT assay in RWPE-1 and WPMY-1 cells. As shown in Figure 9A, treatment with HBX-5 (15.6–1000 μg/mL) for 24 h had no effect on the cell viability in RWPE-1 and WPMY-1 cells. Next, we demonstrated the cell proliferation effect of DHT on RWPE-1 cells and WPMY-1 cells and evaluated inhibitory effect of HBX-5. RWPE-1 cells and WPMY-1 cells were treated with 10 nM of DHT for 72 h and 24 h respectively, with or without HBX-5 (15.6–1000 μg/mL). We investigated significant cell proliferation in DHT 10 nM treated group compared to the control group. However, HBX-5 (15.6–1000 μg/mL) treated groups significantly inhibited the extent of cell proliferation with DHT treatment in RWPE-1 cells. In WPMY-1 cells, treatment with HBX-5 (125–1000 μg/mL) for 24 h had a significant inhibitory effect on the cell proliferation which was stimulated with DHT.

In addition, we investigated the anti-BPH effects of HBX-5 at the concentrations of 50, 100, and 200 μg/mL in DHT-stimulated RWPE-1 and WPMY-1 cells. In this study, in vitro BPH model data suggested that androgen and AR signaling is a common condition, which has considerable impact on promoting prostate cell proliferation. Among numerous androgen-related genes, PSA is a fundamental characteristic of BPH, because it is primarily regulated by AR signaling at the transcriptional level [24]. As shown in Figure 9C, the expression of AR and PSA were significantly up-regulated in response to DHT, whereas pre-treatment with HBX-5 inhibited the expression of AR and PSA.

## 3. Discussion

Many researchers have argued that several mechanisms are involved in the development and progression of BPH. Among them, the development of BPH requires the presence of testicular androgens. Previous studies have reported that hypertrophic prostate tissue has higher dihydrotestosterone activity than that of normal prostate gland tissue [25]. A huge number of studies have been published on the involvement of hormonal environment in prostatic enlargement [26,27]. The enlargement of the organ, which is considered to be one of the important biomarkers of BPH, is also considered to be important for histological diagnosis characterized by proliferation of the stromal and epithelial components [28]. The incrimination of androgen, particularly DHT, in the pathogenesis of BPH forms the basis of the current use of 5α-reductase inhibitors in the treatment of symptomatic nodular hyperplasia. Besides prostatic enlargement and involvement of DHT, administration of testosterone revealed increased protein level of proliferation and abnormal expression of AR and 5α-reductase [29].

A clinical study reported that medical therapies, such as 5α-reductase inhibitors and α1-blockers trigger adverse effects like ejaculatory or erectile dysfunction and decrease libido [30]. In addition, the clinical study rendered a warning that the long term use of 5α-reductase inhibitors and α1-blockers will increase the risk of excessive collagen disposition and fibrosis, which is a dominant factor for the development of BOO and LUTS [31]. As part of the efforts to substitute synthetic medications and minimize the side effects, phytotherapy has been gaining attention. Recently, phytotherapy represented more than 90% of all the treatments prescribed for BPH in Germany and Austria, and its use has increased considerably in the USA [32]. Accordingly, many studies have been conducted to assess the clinical evidence on the alternative treatment for BPH [33]. Meanwhile, herbal medicine has been shown to have the side effects due to incorrect dosage, lacking of standardization and quality control of the herbal drugs, and interaction with conventional dugs. Thus, proper quality control and standardization of raw materials and the clinical trials should be carried out [34].

In this study, HBX-5 from the nine medical plants were prepared: Roots of *Aconiti Lateralis* Radix Preparata, fruits of *Cornus officinalis*, stems of *Cistanche salsa*, seeds of *Psoralea corylifolia* L., above ground of *Dendrobium nobile* Lindley, roots of *Morinda officinalis* Haw, seeds of *Cuscuta japonica*, seeds of *Trigonella foenumgraecum* L., and fruits of *Foeniculum vulgare* Mill. To confirm the constituents of HBX-5, representative chromatograms of the mixed standards and extracts were analyzed. HPLC analysis revealed persistent high contents of psoralen and isopsoralen, which can be explained by the acid hydrolysis of psoralenoside and isopsoralenoside, respectively. Based on the HPLC analysis and previous studies, which reported the anti-proliferation efficacy of psoralen and isopsoralen from *Psoralea corylifolia* L. on the transplanted tumor in nude rats, we hypothesize the anti-proliferation ability of the single compound, particularly from *Psoralea corylifolia* L., and the mixed extract of nine herbs that could potentially reflect BPH suppression effects.

In this study, HBX-5 effectively reduced prostate weight and inhibited the pathological alterations induced by testosterone injection, and the results were comparable to the finasteride-treated group. In addition, a number of analyses have examined the relationship between the endocrine system in BPH and a series of pathological conditions, such as chronic inflammation and abnormal tissue remodeling [35,36]. Testosterone-induced BPH group showed an increase in the serum DHT production and 5α-reductase level and over-expression of AR, whose activation can reflect interaction with endogenous androgens. However, HBX-5 markedly reduced the expression of AR and had significant suppressive effects on the expression of 5α-reductase. Consistent with in vivo study, we found that DHT-treated prostate cell line showed over-expression of AR and PSA.

PSA is an established good marker for prostate cancer but can also be used in the diagnoses of BPH and provides important likelihood to progress [37]. The elevated concentration of PSA is also found in BPH; prostate inflammation and PSA are biomarkers for assessing the prostate volume [38]. As shown in Figure 9, HBX-5 significantly decreased the AR and PSA levels in RWPE-1 prostate epithelial cells and WPMY-1 prostate stromal cells. Wang et al. showed the expression of inflammatory-related enzymes COX-2 and iNOS in the prostate epithelial cell [39]. In addition, previous studies have reported that COX-2 induces a higher cell proliferation and up-regulates the expression of anti-apoptotic Bcl-2 gene, highlighting further correlations between inflammation, apoptosis, and prostate growth imbalance [40]. It has been suggested that the Bax/Bcl-2 ratio is essential for the androgen regulation of apoptosis, and the development of BPH is causally associated with the alterations in Bax-to-Bcl-2 balance and caspase-3 activity [23,41]. Thus, the role of inflammation and apoptosis in BPH cannot be overemphasized. In the present study, it was demonstrated that the administration of HBX-5 reduced the level of COX-2 and iNOS in rats with BPH.

In addition, HBX-5 induced apoptosis reduced the level of Bcl-2, Bcl-xl, and Survivin acted as an upstream regulator of mitochondrial dependent apoptosis. The results indicated that HBX-5 might suppress the growth of prostate cell through its anti-inflammatory and pro-apoptotic effects.

In the BPH model, abnormal prostate growth is related to the activation of proliferation process, and vice versa for the inhibition of the apoptotic pathway, which is induced by excessive androgen stimulation, thereby demonstrating a key role of DHT in the development of BPH [42]. As shown in Figure 4A and Figure 6, we found that testosterone injection induced the thickening of the epithelium and markedly increased the expression of PCNA in the prostate tissue, and administration of HBX-5 reduced the level of PCNA in rats with BPH. PCNA has been shown to be directly correlated with the proliferative state of various tissues, and so, correlates with the development of prostate diseases [43].

In summary, evidences from this study suggest that HBX-5 was able to alleviate the development of BPH due to its anti-inflammatory and pro-apoptotic effects. Based on this study, it is recommended that HBX-5 be further explored as a potential candidate for the treatment of BPH.

## 4. Materials and Methods

### 4.1. Chemicals and Reagents

Testosterone propionate was purchased from Wako Pure Chemicals (Tokyo, Japan). Finasteride, a type II 5-reductase inhibitor, was obtained from Merck & Co., Inc. (Whitehouse Station, NJ, USA). 5α-Reductase 2 and glyceraldehyde-3-phosphate dehydrogenase (GAPDH), and proliferating cell nuclear antigen (PCNA) oligonucleotide primers were purchased from Bioneer Corporation (Daejeon, Republic of Korea), and SYBR Premix Ex Taq was purchased from Takara Bio., Inc. (Otsu, Japan). Antibodies against the androgen receptor (AR; N-20; cat. no. sc-816), inducible nitric oxide synthase (iNOS; M-19; cat. no. sc-650), cyclooxygenase 2 (COX-2; C-20; cat. no. sc-1745), Bcl-2 (C-2; cat. no. sc-7382), Bcl-xL (H-5; cat. no. sc-8392), Bax (B-9; cat. no. sc-7480), Survivin (D-8; cat. no.sc-17779) and β-actin (ACTBD11B7; cat. no. sc-81178) were purchased from Santa Cruz Biotechnology, Inc. (Dallas, TX, USA). Horseradish peroxidase-conjugated secondary antibody was purchased from Jackson ImmunoResearch Laboratories, Inc. (West Grove, PA, USA). Standard samples of Loganin (**4**), echinicoside (**5**), verbascoside (**6**), psoralen (**7**) and isopsoralen (**8**) were purchased from Sigma-Aldrich (Seoul, Korea). Morroniside (**3**) was purchased from ChemFaces (Wuhan, Hubei, China). The purity of each compound was determined to be greater than 95% by HPLC analysis. The chemical sturctures of these compounds are shown Figure 1. HPLC-grade acetonitrile and water were purchased from JT & Baker (Seoul, Korea). All other solvents and chemicals were of analytical or HPLC grade.

### 4.2. Preparation of HBX-5

HBX-5 sample was purchased from Hwapyung D & F co., Ltd. (Seoul, Korea). The composition of HBX-5 produced from the sources of herbs is shown in Table 1. The composition of HBX-5 was as follows (values indicate proportions of each ingredient, expressed in parts per whole): *Psoralea corylifolia* L. (100 g), *Cistanche deserticola* Y.C. Ma (120 g), *Cuscuta chinensis* Lam. (120 g), *Dendrobium loddinesii* Rolfe (80 g), *Morinda officinalis* How (120 g), *Trigonella foenumgraecum* L. (80 g), *Foeniculum vulgare* Miller (40 g), *Cornus officinalis* Sieb. et Zucc. (120 g), and *Aconitum carmichaeli* Debx. (20 g). These nine crude herbs were decocted gently in 10 times of their volume of 30% EtOH for 5 h, filtered, and the powder was spray-dried to obtain an extract at a yield of about 8.9% by weight of the original preparation.

### 4.3. Instruments

Waters e2695 Alliance HPLC system (Milford, MA, USA), equipped with column and sample compartment with temperature control and photodiode array wavelength detector (PDA) (Waters 2998), quaternary pump, autosampler, and on-line degasser was used. Data acquisition, analysis, and reporting were performed using Empower chromatography software (Milford, MA, USA).

### 4.4. Sample Preparation for HPLC

Ultrasonic extraction (USE) was carried out using Wiseclean ultrasonic instrument (WUC-A22H, Seoul, Korea). Powdered HBX-5 (0.5 g, 0.50 mm) was mixed with 100 mL methanol (5 g/L) and treated in an ultrasonic bath (40 kHz, 25 °C) for 1 h. The extract was then filtered through a 0.22 μm PVDF membrane filter. For the analysis of psoralen and isopsoralen, acid hydrolysis was carried out by soxhlet apparatus. Powdered HBX-5 (0.5 g, 0.50 mm) was mixed with 100 mL of 0.5 N hydrochloride (5 g/L). Subsequently, methylene chloride (100 mL) was added, and the mixture was shaken vigorously. Methylene chloride layer was concentrated to dryness at 40 °C under vacuum. The extract was diluted in methanol. The solution was then filtered through a 0.22 μm PVDF membrane filter.

### 4.5. Calibration Curves, Limits of Detection, and Quantification

Methanol stock solution (70%) containing the 6 reference components was prepared and diluted to a series of appropriate concentrations for the construction of calibration curves. Six concentrations of the mixed standard solution were injected in triplicate, and their regression equations were calculated according to Y = AX + B. The results are demonstrated in Table 1. The diluted solution was further diluted to a series of concentrations with 70% methanol for the determination of the limits of detection (LOD) and quantification (LOQ). The LOD and LOQ under the present chromatographic conditions were determined at a signal-to-noise (S/N) ratio of 3 and 10, respectively. LOD and LOQ for each analyte are also shown in Table 1.

### 4.6. UFLC-IT-TOF MS Analysis

The LC analyses were performed on a Shimadzu analytical UFLC system (Kyoto, Japan) consisting of two LC-20AD XR pumps (pump A and pump B), a CTO-20A column oven, a DGU-20A3 degasser, a SPD-20A UV detector, and an SIL-20A XR autoinjector. An ACQUITY UPLC^®^ BEH C18 column (1.7 um, 2.1 mm, 150 mm, Waters, USA) with a column temperature of 40 °C was used for sample separation. The UFLC mobile phases were pumped by pump A (water) and pump B (acetonitrile), respectively; the water phase contained 0.1% formic acid (pH 2.70), and the flow rate was 0.3 mL/min. The on-line chromatogram of HBX-5 was recorded by UV1 at 254 nm in the UFLC system. A gradient elution was programmed as follows: 0–20 min, 5.0–90.0% (*v*/*v*) B; 20–24 min, 90–90% B; 24–25 min, 90–5% B; 25–30 min, maintaining 5.0% B. Injection volume was 1.0 μL. The above UFLC system was connected to a hybrid IT-TOF mass spectrometer (Shimadzu LCMS-IT-TOF, Kyoto, Japan) via an ESI interface. The detector voltage was fixed at 1.67 kV and the curved desolvation line (CDL) temperature and block heater temperature were both maintained at 200 °C. The flowrate of nitrogen as nebulizer gas was 1.5 L/min. For full scan MS analyses, spectra were recorded in the range *m*/*z* 100–1000. Data were processed later by LC/MS solution software (version 3, Shimadzu, Kyoto, Japan), which included a formula predictor.

### 4.7. Animals

Ten-week-old male Sprague-Dawley rats (200 ± 50 g) were purchased from Daehan Biolink Co. (Daejeon, Korea). The animals were housed under conditions in accordance with the guidelines for the care and use of laboratory animals, which were adopted and promulgated by Sangji University according to the requirements established by the National Institutes of Health. All the experimental protocols were approved by the Institutional Animal Care and Use Committee (IACUC) of Sangji University prior to the initiation of any experimental study (IACUC Animal approval protocol #2016-14). The rats were acclimatized to the laboratory conditions for 1 week, prior to initiating the experiment. BPH was induced in the rats by intramuscular injections of testosterone propionate after castration. Briefly, rats were divided into six groups: Group 1—control group (Control, normal prostate with vehicle: 200 μL corn oil; subcutaneous injection (s.c.)); Group 2—rats with BPH group (BPH); Group 3—rats with BPH group treated with 5 mg/kg/day of finasteride; Per os (p.o.) (Fina); Group 4—rats with BPH group treated with 50 mg/kg/day of HBX-5; p.o. (HBX-5 50); Group 5—rats with BPH group treated with 100 mg/kg/day of HBX-5; p.o. (HBX-5 100); Group 6—rats with BPH group treated with 200 mg/kg/day of HBX-5; p.o. (HBX-5 200). rats with BPH groups were injected with testosterone propionate (10 mg/kg/day) alone or along with HBX-5 or finasteride for four weeks except weekends. After 24 h of the last administration, all rats were sacrificed after anaesthetization with zoletil 50 (i.p., 20 mg/kg). Blood samples were drawn from the caudal vena cava and the serum was separated by centrifugation at 4 °C and stored at −80 °C. The entire prostate gland was removed and weighed.

### 4.8. Prostate Weight to Body Weight Ratio

Prostatic tissues were excised, rinsed, and weighed immediately. Prostate weight to the body weight ratio (PW/BW ratio) was calculated as described below.

PW/BW ratio = (prostate weight of each animal of the experimental group/the body weight of each animal of the experimental group) × 1000.

### 4.9. Serum Concentrations of DHT Analysis

The serum concentrations of DHT were determined using commercial ELISA kits (ALPCO Diagnostics, Salem, NH, USA). The assays were performed according to the manufacturer’s instructions.

### 4.10. Histological Analysis

The prostatic tissues in each group were fixed with 4% formalin and embedded in paraffin, and the tissues were cut into 4-mm sections. The sections were stained with hematoxylin and eosin (H&E) for histological examination. Images were acquired using a Leica microscope (Leica DFC 295, Wetzlar, Germany).

### 4.11. Quantitative Real-Time Polymerase Chain Reaction Analysis

The prostatic tissues isolated from each animal were homogenized, and the total RNA was isolated using Easy-Blue^®^ reagent (iNtRON Biotechnology, Inc., Gyeonggi-do, Korea) according to the manufacturer’s instructions. The total RNA was quantified using an Epoch^®^ micro-volume spectrophotometer system (BioTek Instruments, Inc., Winooski, VT, USA). The total RNA from the prostate was converted to cDNA using a high-capacity cDNA reverse transcription kit (Life Technologies, Grand Island, NY, USA). Polymerase chain reaction amplification was performed with the incorporation of SYBR green (Life Technologies). The oligonucleotide primers designed from the rat genome for 5α-reductase 2 were GGC AGC TAC CAA CTG TGA CC (forward) and CTC CCG ACG ACA CAC TCT CT (reverse), for PCNA were CTG CTG GGA CAT CAG TTC GG (forward) and GAT CGC AGC GGT ATG TGT CG (reverse), and for GAPDH, which was used as a housekeeping gene, were TGA TTC TAC CCA CGG CAA GT (forward) and AGC ATC ACC CCA TTT GAT GT (reverse). Reverse transcription was conducted using a thermo cycler (Gene Amp^®^ PCR system 9700, Life Technologies), and the results were expressed as the ratio of optimal density to GAPDH.

### 4.12. Western Blot Analysis

The prostatic tissues isolated from each group were thawed and homogenized using the Pro-prep^®^ protein lysis buffer (iNtRON biotechnology Inc, Kyungki-Do, Republic of Korea). Protein extracts were incubated for 30 min on ice to induce cell lysis. Tissue extracts were centrifuged at 13,000 rpm at 4 °C for 30 min, and the supernatant was transferred to a clean tube. Protein samples (30 μg each) were separated on an 8–12% sodium dodecyl sulphate-polyacrylamide gel. After electrophoresis, proteins were transferred to PVDF membranes. The membranes were blocked in 2.5–5% skim milk for 30 min, and incubated overnight with specific primary antibodies in Tris-buffered saline (TBS) containing 0.1% Tween 20 at 4 °C. Primary antibody was removed by washing the membranes three times with TBS-T buffer and incubated for 2 h with horseradish peroxidase-conjugated secondary antibody (1: 2500) at 25 °C. After washing three times with TBS-T, immuno-detection bands were reacted with ECL solution (Ab signal, Seoul, Republic of Korea) and manifested on a X-ray film (Agfa, Belgium).

### 4.13. Immunohistochemistry

All Immunohistochemistry (IHC) analyses was performed on formalin-fixed and paraffin-embedded samples. Paraffin blocks were sectioned to 7-μm thickness. Then, poly-L-lysine-coated slides were used to promote adhesion of the paraffin-section to the slides, which were then dried. The dried slides were de-paraffinized and endogenous peroxidase exhaustion was performed. Sections were blocked for 1 h with 10% normal goat serum (Gibco Life Technologies, Grand Island, NY, USA), prior to incubation with primary antibody (1:200) overnight at 4 °C. Secondary antibodies (1:100) were used to detect primary antibodies. Diaminobenzidine (DAB, Dako, USA) was used to induce signaling, and hematoxylin was used as a counterstain. Images of the IHC slides were visualized by optical microscopy (ECLIPSE Ni-U, Nikon, U.S.A.) and rendered using NIS-Elements F Ver 4.0. For IHC, AR and PCNA antibodies were used.

### 4.14. Cell Culture and Sample Treatment

The normal human prostatic epithelial cell line RWPE-1 and normal human prostatic stromal cell WPMY-1 were obtained from the American Type Culture Collection (Manassas, VA, USA). RWPE-1 cells were cultured in Keratinocyte Serum Free Medium (K-SFM), supplemented with 0.05 mg/mL bovine pituitary extract (BPE), 5 ng/mL human recombinant epidermal growth factor (EGF) and antibiotic-antimycotic (Gibco, Big Cabin, OK, USA). WPMY-1 cells were cultured in Dulbecco’s Modified Eagle’s Medium (DMEM), supplemented with 1% penicillin/streptomycin and 10% FBS (Gibco, Big Cabin, OK, USA). After 24 h of incubation, the cells were starved for 24 h prior to some of the experiments. Then, the cells were treated with 10 nM DHT for 24–72 h, with or without various concentrations of HBX-5 (50–200 μg/mL).

### 4.15. Statistical Analyses

Results are expressed as the mean ± standard deviation (SD) of triplicate experiments. Statistically significant values were determined using ANOVA and Dunnett’s post hoc test, and *p*-values of less than 0.05 were considered statistically significant using Prism 5.

## Figures and Tables

**Table 1:** Compounds contents of HBX-5.

No.	Analyte	Purity	Contents (mg/g)
(3)	Morroniside	97.0%	5.299 ± 0.530
(4)	Loganin	97.0%	2.547 ± 0.245
(5)	Echinacoside	98.0%	1.958 ± 0.168
(6)	Acteoside	99.0%	0.554 ± 0.004
(7)	Psoralen	99.0%	2.040 ± 0.011
(8)	Isopsoralen (Angelicin)	95.0%	2.495 ± 0.025

No. 3–6: before acid hydrolysis of HBX-5; No. 7,8: after acid hydrolysis of HBX-5. Data is represented as the mean ± SD (*n* = 3).
